# Prognostic Factors in Intra-articular Platelet-Rich Plasma Treatment for Knee Osteoarthritis: A Comparative Analysis of Responders and Nonresponders

**DOI:** 10.7759/cureus.57645

**Published:** 2024-04-05

**Authors:** Masataka Ota, Takayuki Okumo, Atsushi Sato, Reo Nagasaka, Marika Mukunoki, Kanako Izukashi, Jun Oike, Saki Yagura, Takayuki Koya, Koji Kanzaki

**Affiliations:** 1 Department of Orthopedic Surgery, Showa University Koto-Toyosu Hospital, Tokyo, JPN; 2 Department of Orthopedic Surgery, Showa University Fujigaoka Hospital, Yokohama, JPN; 3 Department of Physiology, Graduate School of Medicine, Showa University, Tokyo, JPN

**Keywords:** non-responders, responders, platelet-rich plasma, knee osteoarthritis, prognostic factors

## Abstract

Background: Knee osteoarthritis (KOA) is a chronic joint disease affecting activities of daily living (ADL) and quality of life due to pain and limited range of motion, afflicting a large number of patients worldwide. However, it is difficult to prevent the progression of the disease. Therapeutic strategies for KOA aim to maintain ADL and QOL by alleviating pain or managing locomotive function. Recently, intra-articular injection of platelet-rich plasma (PRP) has been gaining attention. In this study, the clinical results of PRP treatment in our institution were reported and compared between responders and non-responders using patient characteristics and imaging data assessed from plain X-rays and magnetic resonance imaging (MRI).

Methods: Participants in the study were KOA patients with varus deformity assessed as grade 2 or higher in the Kellgren-Lawrence classification who received PRP treatment from January 2022 to November 2023 and were followed up for at least three months. PRP was prepared with 27 mL of blood collected from the patient, and 2.7 mL of PRP was prepared using the PEAK©︎PRP System from DePuy Synthes (Raynham, MA). Intra-articular injections of PRP were performed under echo-guided procedures, and responders or non-responders were determined using the Osteoarthritis Research Society International Standing Committee for Clinical Trials Response Criteria Initiative (OMERACT-OARSI) criteria evaluated by the Japanese Knee Injury and Osteoarthritis Outcome Score (J-KOOS) at three months after PRP injection. The clinical efficacy of PRP treatment for KOA was assessed in this study, and a dichotomous analysis was performed comparing the responder group and the non-responder group using patient characteristics and assessed data from plain X-ray images and MRI to determine prognostic factors for PRP treatment.

Results: The study population included 36 knees with a mean age of 70.6. ± 9.2 years, comprising six knees in men and 30 knees in women. The responder group consisted of 16 knees (44.4%), and the non-responder group consisted of 20 knees (55.6%). J-KOOS subscores at pre-treatment elicited that each subscale in the R group was significantly lower than that in the NR group at pretreatment. A dichotomous analysis for the two groups revealed the distribution of sex and past medical history of hyperlipidemia to be significantly different between the two groups. Multivariable logistic regression analysis showed that the coexistence of hyperlipidemia was the main prognostic factor for the efficacy of PRP therapy.

Discussion: In this study, comparisons were conducted between responders and non-responders to estimate prognostic factors for the efficacy of PRP therapy. Surprisingly, responders to the treatment tended to show lower J-KOOS scores and to have hyperlipidemia. A literature review revealed conflicting reports on prognostic factors for PRP therapy in KOA, highlighting the need for further research.

## Introduction

Knee osteoarthritis (KOA) is a degenerative joint disease that affects the knee joint, causing joint pain, functional impairment, limited range of motion, and muscle weakness, thereby impacting patients’ quality of life (QOL) [1]. Currently, there is no curative treatment available to prevent or halt KOA progression. Standard treatments involve exercise therapy, weight management, and symptomatic relief using nonsteroidal anti-inflammatory drugs administered topically or orally, along with intra-articular corticosteroid or hyaluronic acid injections [2].

Current goals of KOA treatment are aimed at maintaining or enhancing QOL by alleviating pain, improving joint function, and enhancing mobility. Surgical interventions, including arthroscopic surgery, around-knee osteotomy, and total knee arthroplasty (TKA), are considered for patients unresponsive to these treatments. TKA is recommended for severe KOA, with a reported 20-year survival rate of 85% and approximately 80% patient satisfaction rates [3,4]. However, the increasing number of TKA surgeries and associated healthcare costs, estimated to account for 1%-5% of various countries’ gross domestic product [5], raises concerns. Additionally, as TKA poses serious risks, including periprosthetic infection, sepsis, deep vein thrombosis, and pulmonary embolism, with potentially fatal outcomes, it is not universally suitable. Consequently, there is an urgent need to develop treatment strategies aimed at delaying the progression of KOA before it reaches a stage where TKA becomes the sole therapeutic option.

In recent years, biological therapies involving autologous stem cells or bioactive substances derived from patients have garnered attention. Platelet-rich plasma (PRP), prepared by centrifuging whole blood to concentrate platelet-rich fluid containing growth factors for potential tissue regeneration, is one such biologic therapeutic agent [6]. PRP was initially used in the 1980s as a transfusion product during open-heart surgery and in the 1990s for dental bone regeneration. In the 2000s, it was clinically applied in orthopedic surgery for sports injuries, and more recently, it has emerged as a treatment option for KOA [7]. PRP plays a crucial role in maintaining tissue homeostasis by inhibiting chondrocyte apoptosis, promoting bone and vascular remodeling, and stimulating collagen synthesis through growth factors released from activated platelets [6].

PRP therapy for KOA has shown superiority over conventional treatments in various studies, emerging as a novel treatment strategy [8,9]. However, prognostic factors for PRP therapy in KOA remain unclear; thus, they represent a vital area for future investigation. This study aimed to explore prognostic factors for PRP therapy in patients with KOA by comparing patient characteristics and imaging data, assessed using plain X-ray and magnetic resonance imaging (MRI), between responders and nonresponders to intra-articular PRP injection administered at our institution.

## Materials and methods

Prior to conducting clinical research, the retrospective research plan for this study received approval from the institutional ethics committee at Showa University, adhering to ethical guidelines for life science and medical research involving human subjects (approval reference number: 22-149-B).

Targeted patients comprised those who underwent PRP therapy between January 2022 and November 2023, as well as a follow-up period of at least three months. Inclusion criteria included a Kellgren-Lawrence (KL) classification of Grade 2 or higher based on anteroposterior views of plain X-ray images [10], along with varus-type KOA determined using the hip-knee-ankle (HKA) angle based on anteroposterior full-length lower limb plain X-ray images. Exclusion criteria included individuals with infectious diseases; positive serum immunoreactivity for hepatitis B, hepatitis C, or syphilis undergoing treatment or with a treatment history; acquired immunodeficiency syndrome; abnormalities in blood counts (e.g., thrombocytopenia, polycythemia, or anemia of <10 g/dL in hemoglobin)' or those who underwent surgical treatments (such as TKA) after PRP therapy.

At our institution, autologous PRP was prepared following the PEAK©︎ PRP System kit protocol from DePuy Synthes Mitek Sports Medicine (Raynham, MA). Briefly, the blood was separated in the first 60 seconds at a centrifugation force of 2500 G, and then the red blood cells and supernatant plasma were drained from the device in approximately 60-120 seconds at the same force. In total, 27 mL of blood was aspirated from the patient via venipuncture and underwent centrifugation, resulting in the extraction of 2.7 mL of leukocyte-rich PRP into a 5 mL syringe under sterile conditions. After aspirating synovial fluid by inserting an 18-gauge needle into the suprapatellar synovial bursa in the knee joint, the entire volume of PRP was injected, with PRP injections performed by three skilled orthopedic surgeons.

**Figure 1:**
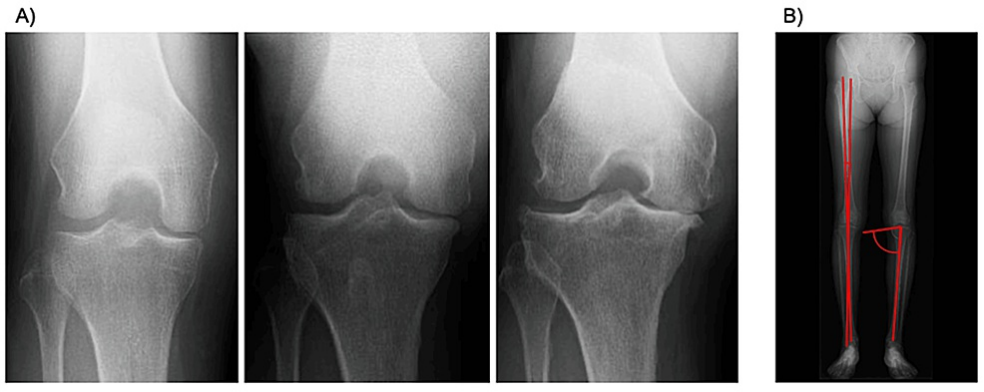
Imaging data analysis with plain X-ray pictures. A) Representative images of the anteroposterior view of the knee joint for Grades 2, 3, and 4 evaluated with Kellgren-Lawrence classification. B) Measurement of the HKA angle and MPTA referring to an anteroposterior full-length lower limb plain X-ray image. The HKA angle is determined by measuring the angle between the line connecting the center of the femoral head and the center of the distal femoral condyle and the line connecting the center of the tibial articular surface and the ankle joint center. MPTA is determined by measuring the angle between the line connecting the medial and lateral edges of the tibial articular surface and the line connecting the center of the tibial articular surface and the ankle joint center. HKA angle: Hip-knee-ankle angle, MPTA: Medial proximal tibia angle

**Figure 2:**
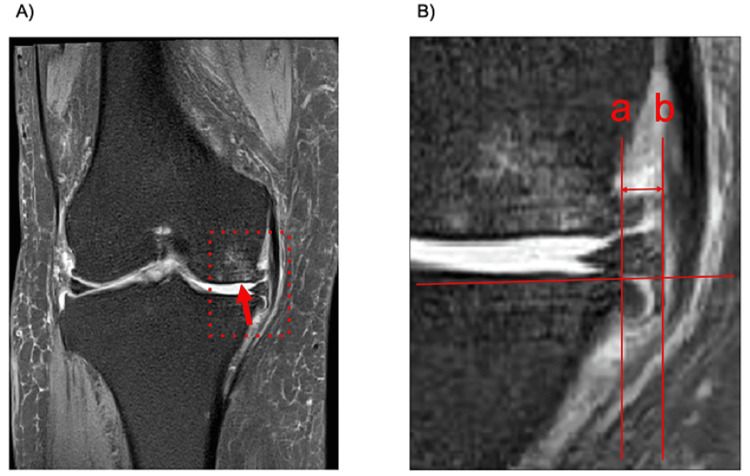
Imaging data analysis with MRI pictures. A) One of the representative images showing the bone marrow lesion in the medial femoral epicondyle (arrow). B) Enlarged image of the area enclosed by the red dotted line in the image (A). Medial meniscus extrusion is determined by measuring the distance between a line (a) perpendicular to the line connecting the medial and lateral edges of the tibial articular surface and passing through the medial edge of the medial tibial joint surface and a line (b) passing through the medial edge of the medial meniscus. MRI: magnetic resonance imaging

Before PRP therapy, demographic data, including age, gender, height, weight, body mass index, and any concomitant medical conditions, were obtained for each patient. Following plain X-ray imaging, KL classification, HKA angle, and medial proximal tibial angle (MPTA) were assessed (Figure 1). KL classification comprises five grades (0-4), with Grade 0 representing an intact condition and Grade 4 indicating end stage [10]. In the present study, Grade 2 was defined as "Moderate," whereas Grades 3 and 4 were categorized as "Severe." Measurement of HKA angle and MPTA referring to an anteroposterior full-length lower limb plain X-ray image. HKA angle is determined by measuring the angle between the line connecting the center of the femoral head and the center of the distal femoral condyle and the line connecting the center of the tibial articular surface and the ankle joint center. MPTA is determined by measuring the angle between the line connecting the medial and lateral edges of the tibial articular surface and the line connecting the center of the tibial articular surface and the ankle joint center. Additionally, the presence of bone marrow edema in the medial femoral condyle and medial tibial plateau and the degree of medial meniscus extrusion were evaluated using MRI imaging (Figure 2). Medial meniscus extrusion is determined by measuring the distance between a line perpendicular to the line connecting the medial and lateral edges of the tibial articular surface and passing through the medial edge of the medial tibial joint surface and a line passing through the medial edge of the medial meniscus. 

The Japanese Knee injury and Osteoarthritis Outcome Score (J-KOOS) was employed for clinical evaluation. The J-KOOS was administered at treatment initiation and during the three-month follow-up appointment. The Osteoarthritis Research Society International Standing Committee for Clinical Trials Response Criteria Initiative (OMERACT-OARSI criteria) was used to assess the effect of PRP treatment [11], with the "pain," "function," and "patient's global assessment" components substituted with the J-KOOS subscales "pain," "activities of daily living (ADL)," and "quality of life (QOL)," respectively [12]. In the final effect assessment, patients were categorized as either a "responder" (R group) or "nonresponder" (NR group).

Candidate predictors were assessed as explanatory variables, with "responder" and "nonresponder" designated as outcome variables based on the OMERACT-OARSI criteria. Explanatory variables underwent normality testing: normally distributed variables were represented by means ± standard deviations, whereas non-normally distributed variables were represented by medians (25th and 75th percentile). Initially, dichotomous analyses of explanatory variables were performed for the R and NR groups. For multiple items showing significant differences, multivariable logistic regression analysis was conducted, considering correlations among the items, to extract prognostic factors for responders/nonresponders regarding PRP treatment. Statistical analysis was performed using JMP Pro (ver. 16.0; SAS Institute Inc., Cary, NC), with statistical significance set at P < 0.05.

## Results

In this study, 36 knees from 36 patients (six in males and 30 in females; mean patient age: 70.6 ± 9.2 years) were eligible for evaluation three months after PRP treatment. The R and NR groups comprised 16 (44.4%) and 20 (55.6%) knees, respectively, as determined through the OMERACT-OARSI criteria. J-KOOS subscores at pre-treatment elicited that each subscale in the R group was significantly lower than that in the NR group at pre-treatment. At three months after PRP treatment, there were no significant differences in all subscales (Table 1). The cutoff values for each subscale were 50.0 points in pain, 58.8 points in ADL, and 25.0 points in QOL.

**Table 1:** J-KOOS subscale scores in each group. Patients were determined to have 16 (44.4%) knees in the responders and 20 (55.6%) in the nonresponders through the OMERACT-OARSI criteria. J-KOOS subscores at pre-treatment elicited that each subscale in the R group was significantly lower than that in the NR group at pre-treatment. At three months after PRP treatment, there were no significant differences in all subscales. *P < 0.05 vs nonresponders evaluated by Student's t-test. ADL: Activities of daily living, J-KOOS: Japanese Knee Injury and Osteoarthritis Outcome Score, OMERACT-OARSI: Osteoarthritis Research Society International Standing Committee for Clinical Trials Response Criteria Initiative, QOL: Quality of life

J-KOOS subscale	Overall	Responders (n = 16)	Nonresponders (n = 20)	P value
Pre-treatment				
Pain	55.6 ± 18.5	45.8 ± 14.6*	63.3 ± 17.9	0.003
ADL	67.8 ± 17.2	57.1 ± 15.8*	76.4 ± 13.2	< 0.001
QOL	36.3 ± 20.9	26.2 ± 13.6*	44.4 ± 22.5	0.007
Post-treatment (3 months)				
Pain	69.5 ± 16.8	73.4 ± 14.5	66.3 ± 18.1	0.211
ADL	76.7 ± 14.1	77.6 ± 12.6	76.0 ± 15.5	0.750
QOL	52.5 ± 21.9	58.6 ± 18.2	47.6 ± 23.7	0.133

Interestingly, dichotomous analysis of various patient profile parameters revealed that gender distribution and comorbidity with hyperlipidemia showed significant differences between the R and NR groups (Table 2). On the other hand, there were no significant differences in the imaging data from X-ray and MRI between the two groups. Multivariable logistic regression analysis showed hyperlipidemia (P = 0.045) as a main prognostic factor for PRP therapy, while sex was not detected as a significant contributor (P = 0.994).

**Table 2:** Dichotomous analysis between the responder group and the nonresponder group. Analysis of various patient profile parameters revealed that gender distribution and comorbidity with hyperlipidemia showed significant differences between the responders and nonresponders (Table 2). On the other hand, there were no significant differences in the imaging data from X-ray and MRI between the two groups. BMI: Body mass index, BML: Bone marrow lesion, HKA: Hip-knee-ankle, KL: Kellgren-Lawrence, MM: Medial meniscus, MPTA: Medial proximal tibial angle

	Overall	Responders (n = 16)	Nonresponders (n = 20)	P value
Physical findings				
Age (yr)	70.6 ± 9.2	73.2 ± 8.4	68.6 ± 9.5	0.138
Sex (Male:Female)	6:30	0:16*	6:14	0.016
BMI	24.8 (22.8, 27.7)	24.1 (22.4, 27.7)	24.9 (23.1, 28.6)	0.691
Synovial fluid (mL)	1 (0, 5.8)	0 (0,2)	2.5 (0, 9.5)	0.220
Comorbidities				
Hypertension (+ : −)	18:18	9:7	9:11	0.502
Diabetes mellitus (+ : −)	6:36	2:14	4:16	0.549
Hyperlipidemia (+ : −)	8:28	7:9*	1:19	0.006
Imaging data (X-ray)				
KL classification (2,3,4)	14, 9, 13	8, 5, 3	6, 4, 10	0.152
Severity of KOA (Moderate:Severe)	14:22	8:8	6:14	0.221
HKA angle (°)	−5.5 ± 3.6	−5.8 ± 3.3	−5.3 ± 3.7	0.622
MPTA (°)	85.4 ± 2.6	84.9±2.4	85.9 ± 2.8	0.260
Imaging data (MRI)				
MM extrusion (mm)	5.6 (2.9, 6.8)	5.5 (3.1, 6.8)	5.8 (2.6, 6.9)	0.750
BML (+ : −)	30:6	13:3	17:3	0.764

## Discussion

In the results of this study, the efficacy rate of PRP therapy for KOA in our facility was under 50% at three months post-treatment. Regarding the J-KOOS, patients in the R group showed lower scores in all subscales at pre-treatment. Furthermore, dichotomous analysis of patient profiles in the two groups revealed that gender and hyperlipidemia as comorbidity might influence the prognosis of PRP therapy. Interestingly, multivariable logistic regression analysis showed that hyperlipidemia as a comorbidity was a chief contributor to PRP treatment.

Several clinical studies have reported on the efficacy of PRP therapy in patients with KOA. Yurtbay et al. [13] conducted a RCT comparing PRP therapy with a placebo group and reported that PRP therapy was more effective over a two-year follow-up period. Additionally, Belk et al. [14] demonstrated the superiority of PRP compared to conventional conservative treatment for KOA, particularly in comparison with intra-articular injections of hyaluronic acid (IAHA). Wang et al. [15] conducted a comparative study with IAHA in patients with early-stage KOA and similarly reported the significant effectiveness of PRP therapy. Furthermore, Huang et al. [16] reported the superiority of PRP over intra-articular corticosteroid injection.

Based on these reports and meta-analyses [17,18], it is presumed that PRP therapy for KOA significantly reduces pain associated with KOA and contributes to improved knee joint function compared to conventional treatment options. Therefore, it is necessary to explore the factors necessary for PRP therapy to be effective in reducing pain and functional disability associated with KOA. The most important finding of this study is that patients in the R group had significantly lower scores on each subscale of the J-KOOS at pre-treatment compared to the NR group and were comparable to the NR group at three months post-treatment. The fact that the degree of patients' discomfort before treatment affects the therapeutic efficacy of PRP therapy may provide a novel perspective on the administration of PRP for KOA patients. In further analysis, cutoff values for each subscale were calculated, which may be an important indicator for providing appropriate information to patients during the pre-treatment medical interview.

Henceforth, several reports comparing responder and non-responder to PRP therapy will be highlighted for further discussion. First, Saita et al. [19] reported results regarding the efficacy rate of PRP therapy stratified based on the KL classification, suggesting that the severity of KOA according to the KL classification may influence the prognosis of PRP therapy. Additionally, while not directly related to the severity of KOA, there are reports suggesting that lower limb alignment and structural changes in intra-articular tissues may impact the efficacy rate of PRP therapy. Kikuchi et al. [20], in a retrospective comparative study involving 72 knees in the responder group and 52 knees in the non-responder group, reported a tendency for knees with advanced varus alignment on simple X-ray full-length images to be classified as non-responders. Furthermore, Toda et al. [21] reported a significant negative correlation between the degree of medial meniscus extrusion on ultrasound imaging and the effectiveness of PRP therapy. Additionally, according to Boffa et al. [22], cases with extensive bone marrow edema tended to show poor efficacy with PRP therapy. As explored above, however, prognostic factors in PRP therapy appear to vary across reports, making it difficult to establish definitive predictors of efficacy. Further investigation is warranted, possibly through conducting comparative trials with placebo or control groups.

On the other hand, one interesting aspect of this study was the finding that sex and hyperlipidemia could influence the efficacy rate in PRP therapy, with men more likely to be in the NR group, and with hyperlipidemia to be in the R group. Xiong et al. [23] reported differences in the concentrations of various chemical mediators in autologous PRP prepared from healthy adults, noting distinctions between men and women in the composition of several factors. Thus, it is undeniable that gender may play a role in these individual differences, particularly when comparing elderly postmenopausal women and elderly men, considering the inherent variations in the amount of chemical mediators in PRP prepared as autologous blood products. It has been reported that hyperlipidemia predisposes to thrombus clot formation, which is a trigger for ischemic heart disease, and that platelets have morphological changes, such as increased fibrinogen binding sites on platelets [24]. The increased platelet aggregation capacity is not fully altered by medication for hyperlipidemia, suggesting that the platelets of patients with the disease are said to be more activated than those of healthy individuals [25]. It is important to have activated platelets in order to obtain a high therapeutic effect of PRP therapy, and the coexistence of hyperlipidemia may have contributed to the therapeutic effect of PRP therapy since platelets are easily activated in this condition. However, it is reported that PRP contains approximately 800 chemical mediators [6], so it remains unclear which of these are the primary bioactive substances responsible for the reduction of pain associated with KOA. Furthermore, the patients in this study were not analyzed for the bioactive substances contained in PRP; thus, no details are available. Therefore, it is difficult to assert that these reports provide sufficient evidence to directly address the validity of this study.

Regarding limitations, firstly, the sample size was insufficient. Considering an effect size of 80%, a type I error rate of 5%, and a type II error rate of 20%, the appropriate sample size should be 50 so that continued research with an increased sample size is necessary. Secondly, the limitation lies in the evaluation of the efficacy within a single facility. A previous report suggests that treatment efficacy in single-center RCTs may be higher compared to multi-center collaborative RCTs [26]. Thirdly, the meta-analysis by Filardo et al. [18] reported that the greatest benefit of PRP therapy was a significantly higher efficacy at 12 months post-treatment compared to other treatments. Therefore, it is also necessary in this study to present follow-up results at 12 months or longer.

## Conclusions

This study investigated prognostic factors for PRP therapy in KOA patients, aiming to identify important predictors of treatment response. The efficacy rate of PRP therapy at three months post-treatment was under 50%, with lower J-KOOS and past medical history of hyperlipidemia influencing treatment outcomes. A literature review revealed conflicting reports on prognostic factors for PRP therapy in KOA, highlighting the need for further research. Limitations include the small sample size, single-facility evaluation, and lack of long-term follow-up data, emphasizing the importance of future studies to address these concerns and provide more robust evidence on the efficacy and predictors of PRP therapy in KOA.
